# News Items

**Published:** 1917-04

**Authors:** 


### News Items.

A machine for surfacing lenses, by Dr. N. Ley Harris, optician, of San Angelo, has recently been placed in operation. The apparatus is said to be the only thing of its kind between Fort Worth and El Paso.

Texas was strongly represented at the conference of Southern health officers which convened in New Orleans, La., March 16th and 17th. Among those in attendance from Texas were: Dr. W. B. Collins, President of the Texas State Board of Health, Austin; Dr. P. W. Covington, the Texas Director for the International Board, Austin; Dr. W. C. Becker, field director of Livingston, Polk county, and Dr. W. L. Culpepper, of Humble.

Efforts to have the American Medical Association, the highest organization of the medicos in the land, to hold its annual meeting in 1918 in Houston are being made by the members of the Harris County Medical Society. An invitation will be extended through the State Medical Association at the next convention of the American Association in New York in June.

The annual meeting of the State Medical Association will meet in Dallas, on May 8th, 9th and 10th. Noted doctors from all over the United States will hold clinics and speak.

### New Hospitals.

The building for the training school for nurses at the Baptist Sanitarium at Dallas will be placed under construction at once. The structure will be five stories and basement and will cost approximately $150,000.

Work on the new $20,000 sanitarium for Forney has already begun. This new sanitarium will be modern in every particular, and Forney can soon boast of having one of the nicest sanitariums in North Texas, and men at the head of it who have a State-wide reputation for surgery.

### Personals.

Dr. C. C. Nash, of Palestine, has recently been appointed chief surgeon of the International & Great Northern Railway.

Dr. W. S. Wysong, of McKinney, has recently been quite ill with pneumonia.

Dr. K. J. Scott, of Fort Worth, recently accepted a position in the King's Daughters' Hospital at Temple.

Dr. J. T. Watson, a well known Dallas physician, was recently injured in an automobile accident.

### Deaths.

Dr. Preston Stewart, a well known East Texas physician, died at Hotel Dieu, in Beaumont, recently, after an illness of three months.

Dr. J. Connor Chisholm died of pneumonia at the Baptist Sanitarium in Dallas recently. Dr. Chisholm was professor of medicine in Baylor Medical College, and was the inventor of what is known as the Chisholm process of refining oil.

Dr. John Cameron, of Wheelock, died at his home February 19th.

